# Affective and Sexual Needs of Residents in Psychiatric Facilities: A Qualitative Approach

**DOI:** 10.3390/bs10080125

**Published:** 2020-08-03

**Authors:** Giulia Landi, Mattia Marchi, Mohamed Yassir Ettalibi, Giorgio Mattei, Luca Pingani, Valentina Sacchi, Gian Maria Galeazzi

**Affiliations:** 1 Department of Mental Health and Drug Abuse, AUSL Modena, Via San Giovanni del Cantone, 23–41121 Modena, Italy; gi.landi@ausl.mo.it; 2 Department of Biomedical, Metabolic and Neural Sciences, University of Modena and Reggio Emilia, Via Giuseppe Campi, 287–41125 Modena, Italy; mattia-marchi@libero.it (M.M.); luca.pingani@unimore.it (L.P.); valentina.sacchi93@gmail.com (V.S.); 3 Department of Primary Care, AUSL Parma, Str. Quartiere, 2/A–43125 Parma, Italy; ettalibi.mohamedyassir@gmail.com; 4 Department of Economics & Marco Biagi Foundation, University of Modena and Reggio Emilia, Via J. Berengario, 51–41121 Modena, Italy; giorgiomatteimd@gmail.com

**Keywords:** sexuality, residential facilities, mental health, sexual health, qualitative research

## Abstract

Background: The affective and sexual needs of psychiatric patients are often under-considered, although they contribute significantly to their general well-being. Such topics are critical for Residential Psychiatric Facilities Users (RPFUs), whose daily life is paced by therapeutic settings. The aim of this paper is to better understand how sexuality and affectivity are expressed by the RPFUs at the Mental Health Department of Modena, within psychiatric residential settings. Methods: Adult RPFUs took part into two audio recorded focus groups. Digital transcripts were analyzed using MAXQDA software in order to perform qualitative narrative analysis, so as to develop a hierarchical code system a posteriori (derived from the data). Results: Eleven participants (eight RPFUs and three investigators) attended the first focus group, and eight participants (5 RPFUs and 3 investigators) attended the second focus group. 175 interventions were analyzed and coded under seven thematic areas: (a) contraception and sexually transmitted disease prevention (*N* = 17); (b) affective needs (*N* = 11); (c) personal experiences (*N* = 61); (d) regulation of sexual relations (*N* = 18); (e) Mental Health Professionals’ (MHPs) openness towards the topic *(N* = 17); (f) MHPs’ responses to RPFUs’ sexual behaviors (*N* = 33); and (g) RPFUs proposals (*N* = 18). The highlighted topics suggest that affective and sexual relations commonly occur within residential psychiatric facilities, even if mental health services often fail to recognize and address RPFUs’ affective and sexual needs as well as to provide effective solutions to manage them. Conclusions: RPFUs expressed a request for support to fulfill their affective and sexual needs and dedicated spaces for sexual activities to relieve their discomfort, while MHPs highlighted a need for awareness, training, and shared problem-solving strategies.

## 1. Introduction

Sexuality is holistically understood as an intrinsic part of well-being that encompasses sex, gender identities and roles, sexual orientation, eroticism, pleasure, intimacy, and reproduction. According to the World Health Organization, sexual health is conceptualized as a state of biological, psychological, and social well-being in relation to sexuality [1]. The expression of affectivity and sexuality also remains a right as well as a need in conditions of vulnerability, such as those experienced by persons with severe mental disorders (SMDs).

Being able to maintain satisfying emotional and sexual relationships may be an indicator of good functioning. Furthermore, enhancing the ability of mental health service users to establish affective relationships reduces internal stigma and is considered a fundamental step towards recovery by many individuals [2].

Despite the growing attention on the rights of people affected by mental disorders, people with SMDs remain frequently stigmatized in many aspects of their lives, including sexuality [3]. In addition, people with SMDs may experience difficulties or challenges related to initiating and maintaining intimate relationships due to the symptoms of the disorder, negotiating sexual safety with reference to prevention of sexual abuses, unwanted pregnancies, and sexually transmitted diseases (STDs), as well as managing sexual side effects caused by pharmacological treatments [3,4,5,6].

Any of these experiences may be detrimental to the individual’s mental well-being and recovery, particularly if the person is not supported to identify, discuss, and address these topics with a trusted mental health professional (MHP). MHPs continue to debate how patients’ right to free expression of affectivity and sexuality can be combined with risk-prevention policies [7]. In the absence of clear guidelines, MHPs often give advice primarily based on personal beliefs about sexuality rather than on evidence [8]. Thus, sexuality and issues related to users’ affective lives appear to be an ongoing challenge to clinical practice in mental health settings [9].

The purpose of this study was to explore the perceived needs of residents of Northern Italy’s psychiatric residential facilities (PRFs) in the areas of affectivity and sexuality.

## 2. Materials and Methods

This study was designed according to the Consolidated Criteria for Reporting Qualitative Research (COREQ) guidelines [10]. Two focus groups (FGs) were carried out [11]. Three investigators attended each FG, and the research was conducted through a neutral approach, where participants could freely express their viewpoint on topics introduced by investigators’ general and open questions about the subject of the study. There was no previous personal or professional acquaintance between participants and investigators.

The two FGs were conducted at Modena East Community Mental Health Centre. All participants were living in PRFs at the Department of Mental Health and Drug Abuse of Modena (Italy), including long-term residential facilities and supported accommodations. The first kind of facilities provide medical psychiatric treatments and rehabilitation programs for people suffering from SMDs in a subacute stage of the illness. In these facilities, health care is provided by MHPs for at least 12 h per day, for a maximum stay of 180 days. The latter facility is comprised of therapeutic communities and shared apartments. Therapeutic communities host a maximum of 12 patients up to 1 year; MHPs’ supervision is provided for at least 6 h per day. Similar duration and kind of support are found in shared apartments, which differ in their maximum capacity, which is up to 6 patients, usually in a chronic stage of illness. Moving forward from long-term residential facilities to supported accommodations, rehabilitation purposes prevail over medical treatments [12]. RPFUs that took part in this study were mostly affected by psychotic disorders (mainly schizophrenia), followed by severe personality disorders and affective disorders (i.e. depressive and bipolar disorder); nearly everybody was taking at least one Second Generation Antipsychotic (SGA) medication.

FGs were audio-recorded. Full transcriptions were then made using Microsoft Word 2016 software. The transcripts were analyzed independently by two investigators (EMY and CC), with the independent supervision of a third investigator (VS), according to the general principles of content analysis so as to develop a hierarchical code system a posteriori (derived from the data). Coding was carried out using MAXQDA 18 software. This codification made it possible to discuss textual fragments and compare them in a systematic way. Therefore, the software setting was not set immediately but after identification of relevant themes using “labels” (e.g., affective needs, regulation of sexual relations, etc.); each label (decided a posteriori while reading the thick descriptions) identifies a fragment of the text that, according to investigators, provides information regarding the aim of the study. The software used for the analysis makes it possible to recall all fragments that were coded with a certain label. Therefore, a certain phenomenon (e.g., “affective needs”) may be explored by using the participants’ words and several points of view. Once all interventions deemed relevant are coded, the second part of the qualitative analysis starts, which is based on the constant comparison of different labels. This process ends when investigators believe that theoretical saturation is reached, i.e., all possible information was extracted as data by means of the coding system. More details concerning qualitative methodology may be found elsewhere. [11,13,14,15,16].

The study was approved by the local ethics committee (reference: 299/16) and was conducted according to the Declaration of Helsinki, Good Clinical Practice principles for medical research, and current regulations relating to the protection and processing of personal and sensitive data (European Regulation n. 679/2016). Prior to commencing each interview, the investigators explained the project to the participants and obtained written informed consent to participate. Participants were first assigned a letter (U as in ‘user’) and a progressive number, and then the transcripts were anonymized.

## 3. Results

Overall, 175 interventions were coded through the code system shown in Table 1.

The first FG lasted 77 min, and the second FG lasted 72 min. A total of 13 RPFUs (8 and 5, respectively) participated. Seven areas were identified and coded (‘main codes’):

1. Contraception and STD prevention.
2. Affective needs.
3. Personal experiences: further expanded into five sub-codes according to the content of the excerpts: friendship, relationship management, sexual intercourse (further divided in masturbation and paid sexual intercourse), unrequited feelings, and emotional sorrow.
4. Regulation of sexual relations.
5. MHPs’ openness towards the theme: further divided into three sub-codes according to the kind of response received from the MHPs: positive, i.e., cases in which RPFUs managed to deal with the topic with MHPs satisfactorily; negative, i.e., cases in which RPFUs found it difficult to deal with the topic with MHPs; and peer-to-peer support, i.e., cases of RPFUs who preferred to deal with the topic with other RPFUs rather than with the MHPs.
6. MHPs’ responses to RPFUs’ sexual behaviors: sub-codes identified were related to the type of response, namely positive or negative. The former encompassed tacit consents and hotels, while the latter collected cases of punitive attitudes to users’ sexual behaviors.
7. RPFUs’ proposals.

Excerpts have been translated from Italian to English by the authors and are provided below to illustrate the different areas. Words added to improve readability are in square parentheses, and the sections removed for brevity are indicated with […].

### 3.1. Contraception and STDs Prevention

Seventeen (17) emerging issues concerned contraception and STDs prevention. According to many of these interventions, both educational programs and dedicated educational meetings concerning sexual and affective needs are lacking. Indeed, some RPFUs stated that they were self-taught, mostly through media, such as the Internet and television:

*‘I am self-taught: with my smartphone, I read on internet about the treatments and the transmission of the [sexually transmitted] diseases, that is how I keep myself updated. There are diseases, which are transmitted only with a complete sexual intercourse, and not with kisses. Well, that is what I know.’*

*(U4, FG 2)*

When asked about the possibility of receiving information on STD and unwanted pregnancy prevention, reactions of RPFUs were discordant. Some argued that they did not see the need for it, while others would be interested. Notably, only one RPFU reported to regularly undergo blood tests for STDs because he is a blood donor, while a female RPFU reported to have felt pressured to take the birth control pill:

*‘Regarding contraception, healthcare professionals agreed with my parents that it was better for me to take the pill. But I did not want, I would love another pregnancy.’*

*(U2, FG 1)*

Desire for parenthood is often strong. People with mental disorders face many challenges in this respect. The potential teratogenic effect of some medications and the prospective fear that their SMDs would interfere with their parental capacity were clearly expressed by a RPFU:

*‘I have been told that pregnancy is not an option. I already had a daughter, who has been entrusted to my parents. If I have another son, he would be taken away from me again. Then, there is the issue of the medication and the malformations in the baby that could happen. I am pointing out that if I desire to have a child, we should agree to stop the therapy or change it, but professionals have been telling me to wait. I am nearly forty, approaching an age when it is no longer easy to conceive a baby, thus I understand that it is no longer possible.’*

*(U2, FG 1)*

### 3.2. Affective Needs

Eleven (11) emerging issues were coded under this theme, including emotional and relational needs perceived by RPFUs. Although the expressions were multifaceted, participation in this topic appeared to be not particularly affected by shame or embarrassment. Antithetic positions, in this regard, were expressed by two RPFUs:

*‘For me, sex with the partner has always been the most important thing in life. I would like to have a woman, a girlfriend.’*

*(User 3, focus group 2)*

*‘I do not feel the need [for sexuality], I do not know why, but actually do not feel it.’*

*(U1, FG 2)*

A common shared belief was that sexuality may be important for improving quality of life and social inclusion but is tough to achieve. Notably, rather than physical contact, RPFUs claimed to pursue a full affective, sexual, and emotional experience:

*‘ […] It is so hard to find someone to trust, someone who calls you to say “good morning”, or asks “how are you?” Nowadays, people are no longer committed in expressing such nice feelings; it would be such a gift to have someone that asks you “How are you?” after a day of work.’*

*(U4, FG 1)*

### 3.3. Personal Experiences

Sixty-one (61) emerging issues were collected under this heading, wherein RPFUs spoke about their personal experiences regarding affectivity and sexuality. Sensitive and personal information was shared. Five sub-codes were subsequently identified: friendship, sexual intercourse, emotional sorrow, relationship management, and unrequited feelings.

RPFUs are or have been sexually active. Often, partners have been living in the same PRF. Difficulties in managing long-lasting relationships were reported besides episodes of jealousy towards friendly bonds with other users:

*‘I have now broken up with my boyfriend. We have been together for ten years, and he lived in the same facility of mine. For staying together [having sexual intercourse], we agreed with doctors to use a hotel. Everybody knew that and allowed it. Now, I have a best friend, he is just a friend, and for me it is enough, yet my ex-partner is jealous of him.’*

*(U5, FG 1)*

#### 3.3.1. Friendship

Another essential theme was the importance of friendships, even if it may be misrepresented and end up becoming a source of suffering and difficult to manage within PRFs:

*‘I had a friendly relation. She is married, and I am single. I appreciate her very much. Once I went to her room, and we had some kind of intercourse. Since that episode, professionals do not let us either talk together, and they keep us faraway, so we had to end our bond; there is not even friendship now.’*

*(U3, FG 1)*

Sixteen (16) emerging issues regarding this sub-theme highlight the importance of friendship for RPFUs with SMDs. Participants perceived friendship as a refuge from loneliness and social isolation they often end up living with due to direct or indirect effects of their disorders, such as stigmatization:

*‘I have a friend. He is my father, my mother, my brother, my sister, my aunt, and my uncle, whom I no longer have. I only have him. It is a very important friendship.’*

*(U1, FG 1)*

Moreover, participants said that the hardest part of building up friendly relations is defining the type of feelings at play, notably whether it is love or friendship.

#### 3.3.2. Relationships Management

Ten (10) excerpts were related to personal stories about the management of relations between RPFUs. To entertain sexual relations within PRFs is often not allowed, and a common reason given for this is the lack of spaces to guarantee privacy. That is why users were sometimes allowed or suggested to have sexual encounters outside of the facility, for example in hotels.

Furthermore, it appeared difficult to maintain relations after the diagnosis of a SMD. These difficulties may lead to the end of the relationship:

*‘I lived with my partner for one year, and everything was well. […] We had a good relationship, but the first time I was admitted to hospital, she came to visit me and said, “I don’t love you anymore. I am leaving you.” It was a blow, from which it was very difficult to recover.’*

*(U6, FG 1)*

#### 3.3.3. Sexual Intercourses

This sub-theme contains 14 excerpts. The sub-code was further split into two ‘micro’-codes: paid sexual intercourses (*N* = 6), and autoerotism (*N* = 4). Some RPFUs reported engaging in sexual acts within the same PRF where they are resident. The majority of these are cases of autoerotism held in the bathroom, according to professionals’ suggestions, to avoid hurting the sensitivity of other users and professionals. However, RPFUs often complained about discomfort for the small and uncomfortable environment:

*‘Sometimes, when I was at home, I masturbated. Here [in the facility], professionals have told me to use the bathroom for doing that. But it is not so comfortable, especially having to stand up or seated onto the lavatory.’*

*(U3, FG 2)*

Two RPFUs reported having paid sexual intercourse and highlighted their viewpoint that these experiences fulfilled a physical need:

*‘Sometimes, I go to the masseuses to let off steam […]’*

*(U3, FG 2)*

*‘It is a mere act [talking about paid sexual intercourse]; there are no feelings, no further purposes, nothing, and that is enough. Moreover, it is physiological.’*

*(U1, FG 2)*

#### 3.3.4. Unrequited Feelings

Under this sub-code, eight (8) emerging issues were collected. All of them underline the disappointment and suffering that ensue from one’s feelings when they are not reciprocated. Hence, there is a need to clarify the type of relationship from the very start:

*‘Already there are problems [with unrequited feelings]. If you are also lovesick, this is really a bad suffering. […] The gist of the issue is that at some point of time, someone will fall in love with someone else. If unrequited, he will suffer.’*

*(U1, FG 2)*

#### 3.3.5. Emotional Sorrow

This sub-code collected 13 emerging issues about the inner emotional suffering that RPFUs have experienced in their lives in relation to their affective needs. Under this sub-code, RPFUs expressed feelings of sorrow related to relational difficulties, including distress derived from SMDs:

*‘I do not even know what love means; I have never had love for me in my life.’*

*(U1, FG 2)*

*‘I had a life so scarce of human relationships that now I consider precious any kind of feeling expressed to me. Even when someone is disappointed by me, actually I am glad, because I can go home saying: “at least, today I had a human contact!”’*

*(U4, FG 2)*

### 3.4. Regulation of Sexual Relations

Eighteen (18) emerging issues were coded under this key theme. Participants mentioned regulations in force within the PRF where they were living. Overall, sexual relations between RPFUs were prohibited within the spaces of the facilities:

*‘Regarding love and affectivity, one of the unwritten rules when you enter the facility is that having sexual intercourses is forbidden. It could happen that two lovers live together in the same facility, however for the sake of respect to other users, it is not possible to have sex.’*

*(U2, FG 2)*

RPFUs agreed that the reason behind the prohibition of sexual intercourse within PRFs is to be respectful towards other guests. A history of two lovers displaying overt affection in the facility, causing discomfort to other users, was brought up as an example:

*‘ […] When X and Y kiss each other, they annoy many. It is not tactful to kiss and touch genital parts in our presence.’*

*(U3, FG 2)*

### 3.5. MHPs’ Openness towards the Theme

Seventeen (17) emerging issues were collected under this code. Here, RPFUs mentioned the responses received from MHPs in terms of dealing with their affective and sexual needs. Subsequently, three sub-codes were identified: positive openness, made up of 12 emerging issues; negative openness, made up of 3 emerging issues; and peer-to-peer support, made up of 2 emerging issues, highlighting that RPFUs sometimes prefer to deal with these topics with other RPFUs rather than professionals.

Most participants reported not having problems in disclosing their affective and sexual lives to MHPs. In order to have an open and trustful discussion with MHPs on these topics, it seems important to be able to choose the right moment for talking. Conversely, shyness and fear of breaches in confidentiality may be obstacles to the building of a supportive and effective discussion:

*‘Yes, I would like to talk about these things [sexual and affective needs], but I am shy and afraid of people. I know that if I could talk about it, I may be helped, but I am not able to do it indeed.’*

*(U8, FG 1)*

#### 3.5.1. Positive Openness

Many RPFUs reported that they had been able to foster a positive alliance with MHPs regarding their affective needs and the management of their sexual relationships and friendships.

Often, MHPs have encouraged users to open up about these topics. This approach seems to be very well received by RPFUs, who reported that they have usually felt better after talking:

*‘Our mental health worker is an open book with us. Indeed, she usually urges us to talk about these things, and I have always been at ease with her. Every time I have told my sexual and affective issues, she has always understood me, and I felt better afterwards.’*

*(U7, FG 1)*

#### 3.5.2. Negative Openness

Even if more rarely, RPFUs have also reported situations in which dialogue with MHPs about their affective and sexual needs has not been satisfactory. Feelings of shame caused by the sensitivity of these issues appears to be the main obstacle:

*‘There are very private issues, and you may feel ashamed to talk of it. It is so thorny to express something that is going wrong in you. It is tough to find both the bravery and the words for going to the professional and saying: “I have sexual problems!”’*

*(U4, FG 1)*

Furthermore, RPFUs may be afraid of the consequences of talking to MHPs about their relationships. This mostly happens when the affective relationship under discussion is between RPFUs, and when the fear is of being forced to interrupt it:

*‘No, [in the case of a friendship between him and another user] I have not asked advices from professionals, because I knew that they, no doubt, would have told me to interrupt this relationship.’*

*(U2, FG 1)*

#### 3.5.3. Peer-to-Peer Support

Due to the aforementioned barriers to the establishment of a proper dialogue with MHPs, sometimes it is easier to seek help from other RPFUs:

*‘We have breakfast, lunch, and dinner together. While we are around the table, it may happen that we chat sometimes of soft topics, and sometimes of serious ones. Some days ago, for example, I have spoken of a relationship of mine with another user, and I have been listened to, understood, and recommended on how it would have been best to behave. I was very happy of that.’*

*(U2, FG 2)*

### 3.6. MHPs’ Responses to RPFUs’ Sexual Behaviors

Under this theme, thirty-three (33) emerging issues were coded, concerning practical management and responses suggested by MHPs when the topic of affectivity and sexuality emerged. According to the type of response provided, two sub-codes were identified: ‘positive responses’ (*N* = 13) and ‘negative responses’ (*N* = 20).

Under ‘positive responses’, it was possible to identify two main attitudes of MHPs: tacit approval and suggesting using a hotel. The former includes three fragments, while the latter is made up of two fragments. Within the ‘negative responses’ sub-code, there are fragments concerning the punishment (*N* = 7) that RPFUs received when they violated the rules concerning sexual activity in force in the PRF where they were living. There are more excerpts regarding negative responses than positive ones, confirming that the affective and sexual behaviors of users are generally perceived to be a problem more than an opportunity.

#### 3.6.1. Negative Responses

RPFUs described situations in which the expression of their affectivity and sexuality was handled unsatisfactorily by MHPs. For example, they did not meet users’ expectations, or provided inadequate or even aggressive responses:

*‘Sometimes, [the MHPs] give you the usual textbook answers, in terms that are too specific to be understood properly. Moreover, they judge [the expression of affection and sexuality] as frivolous and unimportant, and this hurts a lot.’*

*(U4, FG 1)*

*‘Having any relations with Y was absolutely forbidden for me. The evening when [the MPH] caught me in Y’s room, he became angry and shouted at me. He did not hit me, but he treated me very badly.’*

*(U3, FG 1)*

Punitive attitudes carried out by MHPs in response to events not allowed within the PRFs may include verbal reprimands, isolation or separation of lovers, prohibition of the relation, or suspension of pocket money.

#### 3.6.2. Positive Responses

MHPs’ support may be in the form of practical suggestions, psychoeducational sessions, or even proper medical recommendations:

*‘When I had a boyfriend, I usually used condoms. Once it broke, and I told that to professionals. They were not angry at me; they understood that it may happen and accompanied me to the counselling center and helped me with the pregnancy test. They were very precious.’*

*(U5, FG 1)*

Sometimes, RPFUs’ partners live in another PRF, making it difficult to have private encounters or to visit each other. In these cases, MHPs may at times use common sense and show understanding for the situation:

*‘Since I got married, mental health professionals both in the hospital and in the facility were very understanding towards my affective situation. Even if there were men’s and women’s sections, sometimes my husband came to visit me clandestinely, and one night he fell asleep in my bed. Professionals noticed it while they were passing for the night check, but they did not say anything. Afterwards, they said, “You should not do it, but we have turned a blind eye to that.”’*

*(U2, FG 1)*

Finally, two RPFUs described how MHPs have directed them to hotels for spending intimate time:

*‘ [The MHPs] let us go to the hotel and indeed encouraged us to have private meetings.’*

*(U2, FG 1)*

### 3.7. RPFUs’ Proposals

This theme encompassed 18 emerging issues, collecting RPFUs’ opinions, hopes, and proposals for improving the way in which affective and sexual needs are addressed within the facilities where they were living.

Among the improvements, the proposal of offering individual MHP–RPFU listening times may allow them to address the topic more effectively, overcoming the shame that could emerge from the sensitivity of the issue or from the RPFU’s personality:

*‘I would like to propose a little change; that is to reserve professional–user times, in which we can talk about affectivity, sexuality, and, why not, socialization in general, which is an important thing itself. This would help me more, since I am very shy.’*

*(U2, FG 1)*

These moments should be held regularly, for example weekly, and should be structured within proper sessions, as it is for other group meetings:

*‘I would take a day in the week to talk about affectivity within the facility. But it has to be regular so that you can learn more from the professional staff and the other guests, because you may have the chance to better know them.’*

*(U4, FG 1)*

Finally, some RPFUs felt that it would be useful to have dedicated spaces in which they can spend their moments of privacy and enjoy relationships in a fair and respectful way:

*‘I would propose to dedicate a room to these moments, for example the basement room of the facility, so that if sometimes I want to go there, I can do it, either alone or with my partner. This could be the room “to do something”, or a place where I can freely go and stay, without having to stand up in the bathroom, which is disgusting.’*

*(U3, FG 2)*

## 4. Discussion

Sexual relationships are common in PRFs, but a growing literature body confirms that mental health services have failed to address RPFUs’ affective and sexual needs [17,18,19,20,21,22,23]. This is consistent with the findings of our qualitative study, where all the participants expressed affective and sexual needs.

Nevertheless, some MHPs seem to be making efforts to discourage sexual relations rather than providing useful information about sexual activity in their daily practice. This is contrary to a holistic, recovery-oriented approach to care, where sexuality and intimacy are important dimensions of people’s overall well-being and recovery [6,24].

Most participants perceived sexuality as a private, sensitive topic, and this was perceived as a barrier to communication about their sexuality. An open and non-judgmental attitude from MHPs may help overcome shyness and feelings of shame. In this regard, lack of MHPs’ training in the field of affectivity and sexuality can make them less willing to listen to RPFUs’ problems in these areas, ultimately discouraging RPFUs from talking for fear of consequences and undermining the therapeutic alliance [25]. Risk-taking sexual behaviors are not addressed by specific information and educational initiatives. This is in line with the literature, which states that patients’ sources of information are not always appropriate or reliable [19,26]. With respect to contraception, the testimony of a RPFU wishing for pregnancy but being discouraged by family and MHPs is worth of mentioning. Similar episodes have been previously reported [27]. In the current study, participants expressed desire for parenthood and emphasized the emotional aspects of their daily relationship with a loved one. The problems encountered in maintaining long-term relationships within the PRFs, where sexual intercourses are generally forbidden and no spaces are dedicated to intimate encounters, represent long-standing challenges. Yet, sexual intercourse often occurs regardless of the imposed rules, inducing patients to pursue relationships in unsuitable settings, hiding in secluded areas where sanitary conditions may be precarious or even performing sexual acts in common areas, causing discomfort to other residents and staff [23,28]. Participants in FGs also reported both paid sexual intercourse and autoerotism. The responses to these behaviors seem to depend on individual staff members’ sensitivity and background, consequently resulting in somewhat idiosyncratic and incoherent reactions [29]. In our qualitative research, it is notable that remarks about MHPs’ negative responses are more frequent than remarks about their positive ones, suggesting that RPFUs’ affective and sexual behaviors are generally judged negatively. Conversely, MHPs who have managed to build an open dialogue with RPFUs seem to have positively influenced RPFUs’ habits, encouraging romantic or friendship relationships, and providing potentially therapeutic support. This kind of support can amount to giving useful practical suggestions (e.g., suggesting couples to book hotel rooms for intimate encounters), offering psycho-educational sessions, or even imparting actual medical recommendations. Similar positive experiences were reported in literature [24,30].

Finally, our “RPFUs’ proposals” section clearly demonstrates RPFUs’ wishes for a change in their living facilities, requesting spaces where they can display affection and express sexuality with adequate privacy, and regular educational sessions on the topic of affectivity, institutional rules, and STD prevention measures within PRFs. These requests are coherent with both the users’ and MHPs’ expectations highlighted in our recent literature review, emphasizing the users’ desire to express and be respected and supported with regard to their affective and sexual needs [31].

### Limitations

RPFUs who volunteered to participate in this study felt comfortable enough to talk about sexuality with the investigators and reflect on this topic specifically in relation to their lives. Clearly, their perceptions might differ from those who did not participate. All participants were based in the same Italian city (Modena), so other locations may be differently resourced, and users in those contexts might approach sexuality and sexual concerns in different ways. Despite these potential limitations, our results overlap with reports of studies from other locations (e.g., [32,33]). Further research could adopt purposive sampling of RPFUs living in PRFs in other Italian areas, both rural and urban, in order to confirm this study’s findings.

## 5. Conclusions

This study confirms that, regardless of the presence of a SMD, RPFUs living in PRF often have unmet affective and sexual needs. An open and proactive attitude from MHPs about this topic seems to foster a positive therapeutic relationship in which RPFUs feel supported in their quest to fulfill these needs. The lack of dedicated spaces for intimate encounters or sexual activities within the PRF is a long-standing problem, which needs to be addressed. Validated tools to assess the quality of care in supported accommodations and how residents’ sexual health or privacy (e.g., QuIRC-SA [34]) are promoted may represent a first step towards improvements in this field. 

Most of the participants in this study reported barriers in fulfilling their affective and sexual needs, and the reasons for this are directly linked to their SMD. Users living in PRFs may benefit from the support of MHPs to satisfactorily address these needs. Shared decision-making in this matter appears to be welcomed by RPFUs, thus suggesting that reserving dedicated times and spaces to talk about affectivity and sexuality within the PRFs appears worthwhile. To achieve real improvements in this area, training professionals in order to foster a more positive attitude towards sexuality and its expression, while also safeguarding vulnerability, appears to be of utmost importance.

## Figures and Tables

**Table 1:** Code System and Number of References for Each Theme Emerging from the Full Transcription (Key Themes in Bold, Sub-Themes in Normal Font).

Codes	Number of References
**1.** **Contraception and STDs prevention**	**17**
**2.** **Affective needs**	**11**
**3.** **Personal experiences**	**61**
3.1. Friendship	16
3.2. Relationship management	10
3.3. Sexual intercourse	4
3.3.1. Masturbation	4
3.3.2. Paid sexual intercourse	6
3.4. Unrequited feelings	8
3.5. Emotional sorrow	13
**4.** **Regulation of sexual relations**	**18**
5. MHPs’ openness towards the theme	17
5.1. Positive openness	12
5.2. Negative openness	3
5.3. Peer-to-peer support	2
**6.** **MHPs’ responses to users’ sexual behaviors**	**33**
6.1. Negative responses	13
6.1.1. Punishment	7
6.2. Positive responses	8
6.2.1. Tacit approval	3
6.2.2. Hotel	2
**7.** **RPFUs’ proposals**	**18**

STD: Sexually Transmitted Disease; MHPs: Mental Health Professionals; RPFUs Residential Psychiatric Facility Users.
